# Changing hearts and minds: examining student nurses’ experiences and perceptions of a general practice placement through a ‘community of practice’ lens

**DOI:** 10.1186/s12909-018-1182-6

**Published:** 2018-04-05

**Authors:** Robin Lewis, Shona Kelly

**Affiliations:** 1 Department of Nursing and Midwifery, Sheffield Hallam University, S10 2BP, Sheffield, UK; 2 Faculty of Health & Wellbeing, Sheffield Hallam University, Collegiate Crescent, S10 2BP, Sheffield, UK

**Keywords:** General practice, Workforce, Placements, Nurse education, Career choices

## Abstract

**Background:**

The recent UK Government paper ‘Five year forward view’ describes the need to move much patient management from secondary to primary care, and this will require a significant increase in the numbers of General Practice Nurses (GPNs). Until recently, there has been no clear recruitment strategy to address this. There have however been a number of proposed solutions to address the impending GPN recruitment crisis and to increase the numbers of new GPNs in post. General Practitioners (GPs) working in the Advanced Training Practice Scheme (ATPS) have been commissioned by Health Education England to provide placements for student nurses. This paper reports upon the findings of a study evaluating the South Yorkshire ATPS network in relation to nursing students’ perceptions of general practice as a placement and a potential career option post-graduation.

**Methods:**

Data were collected using semi-structured interviews with 18 nursing students. Qualitative data analysis used a framework approach and themes were cross-checked within the team. The research had ethical approval and anonymity and confidentiality were maintained throughout.

**Results:**

Using the Communities of Practice (CoP) framework as a theoretical lens, two main themes emerged from the data: ‘Myths and misunderstandings’ outlined some of the misconceptions that abounded in the absence of an established CoP in general practice. These included perceptions of what constitutes a ‘good’ placement, an apparent lack of relevant content in the curriculum, and the widespread use of social media by students as a means of information gathering. ‘Changing hearts and minds’ referred to the need to positively influence the culture within general practice by addressing some of the longstanding myths. Through the fledgling CoP, the students’ perceptions of the GPN role in particular were positively revised, as was the prospect of a career in general practice upon graduation.

**Conclusions:**

The CoP that is emerging through the ATPS placements appear to be gradually changing the socio-cultural landscape within general practice by enabling student nurses to experience the reality of life in general practice nursing, and to view the GPN role as a viable career option upon graduation.

**Electronic supplementary material:**

The online version of this article (10.1186/s12909-018-1182-6) contains supplementary material, which is available to authorized users.

## Background

There is much reference in the healthcare media to a developing ‘workforce crisis’ in general practice [1]. The Health Education England (HEE) report into primary care [2] and the Royal College of General Practitioners (RCGP) Practice Forum Report [3] both concluded that in some parts of the UK General Practitioner (GP) partners were finding it increasingly hard to recruit GP trainees and to replace those GPs who were opting to retire early. At the same time, the United Kingdom (UK) National Health Service (NHS) England’s ‘Five Year Forward View’ [4] outlined large scale plans to move more health services into the community. It is generally acknowledged that if general practice is to be able to meet this increasing demand in the future, the necessary workforce resources must be put in place to support that transition [5]. The number of older people with multiple, complex long-term conditions is set to grow from 1.9 million in 2008 to 2.9 million by 2018, with the majority of these individuals being managed in primary care [6].

A substantial proportion of the chronic disease management in primary care is now provided entirely by GPNs, and the delegation of this type of work from GPs to GPNs has placed a much greater emphasis upon the importance of the GPN role [7]. Indeed, the evidence shows that GPNs make a significant financial contribution to general practice income through meeting Quality and Outcomes Framework (QOF) targets, particularly in relation to chronic disease surveillance and management [8, 9].

### The importance of placement learning

Currently placement learning makes up 50% of the pre-registration nursing programme, and this equates to 2300 h of learning over three years [10]. It has been argued [5] that at least part of the forthcoming GPN recruitment crisis relates to the dearth of general practice placements available for student nurses in the UK. Moving student placement capacity from secondary to primary care is in line with the philosophy of the Five Year Forward View, and very much reflects the ‘direction of travel’ for future workforce needs. However, unlike medicine, there is no established culture of student nurses spending time on placement in general practice, and this means that many student nurses do not know what general practice nursing is or what it has to offer [11]. This situation clearly needs to be addressed, and one practical way to do this is to increase the number of clinical placements for student nurses within general practice. However, a RCGP survey [12] noted the lack of student nurse placements in general practice, exacerbated by a lack of GPNs with mentorship qualifications. These findings were borne out by the QNI survey [7] which found that only 27% of GPs currently offered placements for undergraduate nursing students, compared to 61.5% offering placements to undergraduate medical students. Most general practices are used to offering placements for student doctors and GP trainees, and the cultural shift required in offering placements for student nurses is significant.

### Placements in general practice

In recent times, there have been a number of proposed solutions to the dearth of student nurse placements in general practice, and to positively influence the developing GPN recruitment crisis [1, 5, 13, 14]. In a number of areas within the UK, GPs have been specifically commissioned to provide placements for student nurses. These schemes are funded by HEE and have been variously known as Community Education Provider Networks (CPENs) or Advanced Training Practices (ATPs), and are part of the wider National Training Hubs Initiative (NHTI) [2]. The published literature on the subject indicates varying degrees of success for the other models, and it also shows that many of the NTHI schemes throughout the UK have been small-scale ‘pilots’ (e.g. [13]), and many appear to have ended once ‘the money ran out’. It is evident that the lack of a truly sustainable model which will ensure continuing ‘buy in’ from GPs, Commissioners and partner HEIs has been one of the factors in this perceived lack of success [5, 13, 14].

Within other professions such as medicine, there are a number of factors that have been identified as crucial to informing students’ future career intentions. For example, there is evidence that GPs play a key role in influencing medical students’ attitudes to general practice as a career [15, 16]. The importance of early exposure to positive role models has been identified as an important factor in this regard. The students in Parker et al. study [15] were positively influenced when they were made to feel part of the team, involved with patient consultations, allowed to carry out practical procedures under supervision, and witnessed what they perceived as good medical practice. While the students reported negative comments about general practice by other colleagues, these had a lesser role in influencing their perceptions of general practice when compared with their own positive experiences. Clearly, many of the factors that positively influenced the medical students were therefore under the control of the GPs themselves. Whilst there is much evidence [15, 16] to help us understand students’ career intentions in disciplines such as medicine, there is currently a dearth of theoretical evidence to help us to better understand this process within the context of student nurses’ career aspirations.

### Communities of practice (CoP) as a theoretical construct

If we are to gain a better insight into how learning takes place within general practice, there is a need to develop a theoretical framework through which to view these processes. The theoretical construct used in this study is based upon the community of practice (CoP). This framework is constructed using Lave and Wenger’s ideas on situated learning in practice communities [17–19], and how they function. According to Lave and Wenger, a community of practice (CoP) is a socially situated, practice-based approach to learning, in which the learning that takes place is viewed in terms of a series of collective, relational and social processes (as opposed to an individual, solitary process of knowledge acquisition). It involves a complex relationship between novice(s) and expert(s), being socialised into the practice and developing an identity within that practice community.

As Chandler and Fry [19] note, a strong learning community promotes interactions and work-based relationships based upon mutual trust and respect. The successful CoP is characterised by having a shared domain of interest, a community that pursues that shared interest and the sharing of knowledge, information and resources. It is argued that the CoP enables the development of positive personal relationships and ways of interacting, together with a mutually constructed sense of identity.

By adopting a socially constructed approach to learning, learning is seen as much more than simply the acquisition of knowledge. Therefore, by using the CoP as a theoretical lens through which to view the students’ general practice placement learning, we may gain a better understanding of the processes by which the nursing students’ views and perceptions are formed. This will enable us to address the key aims of the study, which are outlined below:

#### Aims and objectives

The overarching aim of the study was to explore the students’ perceptions of their placement in general practice. There were a number of specific objectives:

1. How do students prepare for their clinical placements?
2. What factors affect perceptions of general practice as a clinical placement?
3. What factors affect perceptions of general practice as a career choice?

## Methods

### Context

The South Yorkshire ATPS was one of the first of the Practice placement networks to be formed, and has been in existence since 2009 [2]. By providing sustainable placements that offer students an in-depth experience of general practice, this exposure to general practice and specifically the role of the GPN enables the students to develop the skills required to work effectively in a general practice setting. The overarching philosophy of the scheme is to promote lasting cultural change in general practice. Through the ATPS, GPs are supported to ‘grown their own’ GPNs by recruiting new graduate nurses. As the ATPS becomes fully embedded, the idea of ‘growing your own’ is beginning to change the prevailing culture within general practice. Partner HEIs such as SHU work closely with the GPs to provide support for the students (proto-GPN) through the ATPS placements and the recruitment of new graduates into a GPN post (neophyte GPN) and afterwards (emergent GPN) through HEE-funded preceptorship initiatives such as the ‘GPN Ready’ scheme (2).

### Recruitment

A convenience sample of students was recruited from the population of SHU students from all cohorts who had experienced a placement in general practice. Participants were invited to take part in the study via a personalised email which provided information regarding the study. In total, 18 students agreed to participate in an interview.

### Data collection

Data collection was carried out using semi-structured interviews with an interview schedule based upon the findings from a rapid review of the existing literature Additional file 1. There were seven questions in the interview schedule, and these related to the students’ experiences of general practice as a placement. The questions were used as prompts for the interviewer and as a framework to guide the dialogue. The interviews were conducted by a member of the study team and took place at a date and time of the participants’ choosing. With the participant’s consent the interviews were digitally recorded, with each interview lasting approximately 15–20 min. Data collection continued until saturation was reached.

### Data analysis

The raw interview data was transcribed and cross-checked within the team for accuracy. Once it had been cross-checked, the data was analysed using ‘Quirkos’^©^ data analysis software. Data analysis followed the UK National Centre for Social Research ‘Framework’ guidelines [20]. The approach involves the systematic processing, sifting, charting and sorting of interview material into key issues and themes. Its advantage is that it permits both within- and across-situation comparisons and allows the integration of existing knowledge from previous research and policy into the emerging theoretical analysis. All the transcripts were analysed independently by four members of the research team and the interpretation of data was also cross-checked within that team. Verbatim quotes from the participants’ were used throughout the process to enhance the link between the student’s experiences on the one hand and the analysis of the data on the other [21].

### Ethical approval

Ethical approval for the study was obtained from the SHU Faculty Research Ethics Committee. SHU Research governance protocols were adhered to throughout the study. All data was anonymised to maintain confidentiality and to ensure that no individual could be recognised in any subsequent report. Paper based data was kept securely in a locked drawer and electronic data and information was kept on a password-protected computer on a network storage system that adheres to Home Office Standards of Data Security. This data will be kept for a minimum of seven years in accordance with SHU guidelines. All participants provided written informed consent, which was verbally reaffirmed immediately prior to the recording of the interview.

## Results

Using Lave and Wenger’s concept of Communities of Practice [18, 19] as an interpretive framework to guide and inform the analysis, there were two main themes that emerged from the data. The first theme of ‘myths and misunderstandings’ relates to the students’ preparation for their general practice placement, which is seen as life before the creation of a new CoP. The second theme relates to the students’ perception of what they experienced, which is seen in terms of the establishment and maintenance of the new CoP. Since general practice is a relatively new placement, and the placement demographics are unusual (small, geographically and socially isolated teams), different CoPs may take some time to become established. The concept of CoPs in healthcare settings is not new, but has gained much traction in recent times as a means for enhancing knowledge exchange and improving practice [19].

### Theme 1: Myths and misunderstandings

#### First steps towards creating a CoP for general practice

The often piecemeal introduction of the general practice placements has provided a number of challenges for staff and students, and these were reflected in some of the students’ comments. Although the ATPS network is now in its eighth year, it has taken a significant amount of time for the general practice placements to ‘come on board’ in sufficient numbers to have any significant impact upon the students. The absence of an established CoP for general practice meant that as the ATPS approached a sustainable ‘critical mass’ of general practice placements, there was a need to challenge the students’ perceptions of what constitutes a ‘good’ and ‘bad’ clinical placement. Myths and misconceptions (largely negative) about general practice abounded amongst the students, so that the prospect of a placement in a GP surgery was not viewed with wholehearted enthusiasm. Traditionally the majority of the students’ placements have been in hospitals, and the well-established frame of reference in nurse education means that most students still want to work in a hospital ward environment. The well-established hospital CoPs meant that inevitably the students want (and expect) their placements to reflect this perception. As this student noted:

*“I have to confess I w’sn’t looking forward to it* [the placement] *at all I wanted another* [hospital] *ward really… it wa’ completely different though to what I were* (sic) *expecting…”*

(Interview S3)

The need to establish a CoP for general practice was clearly highlighted by the absence of accurate pre-placement information on general practice. This situation was further exacerbated by a perceived lack of general practice content within the undergraduate curriculum. As this student noted:

*“I had no real idea what to expect and no real idea what the nurses did* [there]*… it were* (sic) *alright though and I wish we’d had a bit more on it before we went…”*

(Interview S16)

There was an emerging consensus that the general practice placements needed a much better ‘press’ generally, and that the curriculum needed to catch up with these new developments. This student commented:

*“It’s almost as if it’s* [general practice] *been forgotten here… We don’t do much on it at all at Uni… I think that we should do more on it in lectures…”*

(Interview S7)

This student echoed the perceived lack of primary care content in the curriculum. An embryonic CoP was developing, and the shared learning with regards to chronic disease management was becoming evident:

*“… Now I’ve done one* [GP practice placement] *I think you need to learn about doing clinics an’ that… you need more on what happens to people outside of hospital with long term conditions and stuff like that… what they do normally”*

(Interview S6)

#### Preparation for practice and the use of social media

From a CoP perspective, the means by which students prepared for their general practice placements was an area of interest. As one of the largest nursing and midwifery departments in the UK, the logistics of communicating effectively with students regarding their placements is a challenge in itself. The students are advised to access the online placement profiles that are available, which give them factual and demographic details regarding the placement such as the contact person, phone number and working patterns.

For the majority of the hospital-based placements, there are already well-established CoPs, in which there appears to be a shared perception of placements that has developed over time. Within the ‘accepted tradition’ of student experience, acute wards are seen as ‘busy’ and ‘heavy’ but are an essential rite of passage, non-ward placements are seen as ‘dull’ and not ‘proper’ nursing and community placements are regarded as ‘a bit of a rest’ after a busy, acute ward placement. This perception was borne out by a number of the participants in the study. A number of the students also stated that individual clinical areas have their own reputations, both good and bad. Anecdotally these reputations are acquired and propagated through ‘word of mouth’ and the increasing use of social media.

The perceived lack of preparation for the new general practice placements meant that students turned to other more accessible but less reliable resources to prepare themselves. In the absence of an existing CoP to provide a clear frame of reference, the students described using both Facebook^©^ and WhatsApp^©^ to obtain information from other students about their forthcoming placements. The comments shared on social media sites were seen by the students as an accurate representation of a placement, but clearly may not give a balanced viewpoint. As we are well aware, the issue is that social media is open to abuse in the broadest sense of the word. The information provided on social media is entirely dependent upon the perspective of the individual making the ‘post’, and whether or not they ‘have an axe to grind’. In fact, there were both positive and negative views expressed about general practice placements on social media. On the positive side, the ability to contact other students (who they did not necessarily know) who had experienced a general practice placement was seen as very useful.

*“I checked on our group’s Facebook*^©^ *page and found someone had been on one* [a general practice placement] *and she said it were* (sic) *great* […] *they let her do all sorts of stuff and do clinics and that… she really enjoyed it in the end…”*

(Interview S2)

However this was not always the case, as this student attested:

“… this one girl had put some really s*** comments on the Facebook ^©^ *page about the practice and it really put me off … she really hated it and said it were boring an’ they wouldn’t let her do ‘owt… it did affect me when I first went there”*

(Interview S6)

From an organisational perspective, the opportunity to positively influence the embryonic general practice CoP should not be underestimated. Once a placement’s reputation is established, it is extremely difficult to change the students’ perceptions.

### Theme 2: ‘Changing hearts and minds’

There is a great deal of existing literature on the subject of placement learning. The principles for the provision of successful placement learning are well-documented; however the development of a successful CoP within general practice is more complex. It is predicated upon the establishment of positive and mutually beneficial relationships between student and GPN, and the development of work-based identities. It is apparent that the majority of the students in the study viewed their general practice placement in a favourable light. The experience was generally seen as a positive one, in that it gave the students an insight into general practice as a whole, and the role of the GPN in particular.

#### Establishing the new CoPs

The newness of many of the placements meant that the traditional ‘rules of engagement’ for students took a while to become established and understood. The lack of familiarity on both sides meant that the co-participative nature of the relationship between the student novice and GPN mentor was crucial in enabling the students to gain an understanding of the GPN role within the context and culture of general practice. This process was identified as an essential part of establishing the CoPs. As this student noted:

*“My mentor was great* […] *she had me with her all the time and after a while she let me take on a bit more* [responsibility] *and do a bit more* [like] *running the clinics which was really good and really helped my confidence* […] *the clinics were really interesting and I learned a lot about how important it is for patients to go to the clinics”*

(Interview S8)

The students reported that they were, in the main, very well-integrated into the practice culture, and were provided with many opportunities to both observe and participate in high quality practice-based learning. The social context of general practice, of small, socially isolated teams with a shared sense of purpose often translated into a ‘family’ type of atmosphere. In this quote, the student articulates the developing sense of community.

*“The whole team were great and really friendly but she* [the mentor] *was brill’…* [She was] *knowledgeable, approachable, friendly and very, very patient with me. She taught me loads* […] *she ran her own clinics and the patients seemed to really appreciate her* […] *we really got to know each other and she trusted me to do stuff which was brilliant…”*

(Interview S3)

One of the prerequisites for a successful CoP is the development of relationships based upon respect and trust, and the use of the word ‘trust’ in this quote would indicate that this particular CoP was beginning to develop a positive, mutually beneficial working culture.

#### Perceptions of the GPN role

Having experienced the GPN role within its correct social context, student perceptions were clearly being changed. The social construct of general practice was being positively influenced and the role of the GPN clearly had appeal for the students. This mature student commented:

*“I think it’s an excellent opportunity for us students to go into a GP and see what they do there, ‘cause I realise now that it isn’t boring or a soft option or anything like I thought and they do a lot more interesting stuff… I could see myself working there you know…”*

(Interview S8)

The GPN role, particularly the roles of the more senior, experienced GPNs appeared to be something of a revelation to the students. It was apparent that prior to their placement, few of the students were overly familiar with what a GPN actually did or how you became one. This student made this rather tongue in cheek comment regarding the perception of GPNs:

*“I thought practice nurses were like older* (sic) *like me mum (don’t tell her will you) and didn’t do very much ‘cept sit around, drink tea and vaccinate babies…”*

(Interview S9)

However, the autonomy afforded by the GPN role and the quality of the ‘face to face’ patient contact were highly valued. The opportunity for independent working provided by the GPN role, particularly in the management of long term conditions was one of the reasons offered by the students as a reason for wanting to enter general practice. The autonomy of the role and the prospect of regular one-to-one patient contact, together with the opportunities to influence individuals’ lives for the better were also highlighted as being extremely desirable. As the CoP starts to become established, there was clearly an appreciation of both the breadth and autonomy of the GPN role within the general practice team. As this student noted:

*“My mentor could sort out her own working days* […] *she had her own clinics to do but she still worked as part of the GP team but no-one bossed her or told her what to do* […] *I really liked that…”*

(Interview S5)

The ability to spend ‘quality time’ with their patients was highlighted as an exemplar of the GPN ‘getting it right’ clinically. As this student remarked:

*“X* [the GPN] *had her own patients* […] *she was very knowledgeable about their conditions and the medications they were taking and about them as people* […] *she had time for them and I really liked that”*

(Interview S14)

As the CoP became embedded into the social ‘fabric’ of the practices, the students commented upon the opportunities for situated learning that were available. They were able to practice and consolidate a wide variety of existing technical and non-technical skills, and to learn and develop important new skills such as the management and surveillance of people with long term conditions.

*“She* [the GPN] *ran her own clinics and could prescribe medications* […] *she knew ever such a lot about it* [COPD] *and the medications the patients were on* […] *she was able to spend time with them and they really seemed to appreciate it…”*

(Interview S3)

A number of these thoughts were echoed by this student. Looking through the socially constructed lens of a CoP, she summed up very succinctly the ‘added value’ of the placement as a learning experience:

*“Being with her* [the GPN] *taught me all about people with long term conditions* […] *what questions to ask and when and how to deal with any problems* […] *after a while we did the clinics together and she let me do some of the talking… she showed me how to take a history too…”*

(Interview S4)

#### The longer term influence of the CoPs

As already noted, the dearth of established CoPs for general practice meant that myths and misconceptions abounded amongst the students that were interviewed. The perception that general practice was not a ‘suitable’ environment for a new graduate nurse was widespread and firmly embedded. This student raised one of the more persistent perceptions of general practice that needed to be addressed through the CoPs. The idea that nurses needed experience prior to applying for a post in general practice was still prevalent. When asked, this student responded to me with a question of her own:

*“Can you actually work there as a newly qualified* [nurse]*? I don’t think you can you know… you definitely need to do a ward first”*

(Interview S11)

This student also noted:

*“I thought why are we doing it* [the placement] *when we can’t get a job there? They don’t take newly qualifieds* (sic) *there do they? So what’s the point?”*

(Interview S5)

For a significant proportion of the students though, their experience of the general practice environment was seen as having had a positive impact upon their future career intentions. Having begun to contribute to the cultural shift that was taking place through the CoP, the prospect of a career in general practice on graduation was becoming a reality. As one student noted:

*“You know I’d never thought about it* [a career as a GPN] *before but I certainly will now… I like the fact that you can be your own boss, sort yourself out and see your own patients and that…”*

(Interview S12)

As this student also commented, the main ‘selling point’ for the GPN was both the variety of roles and the new possibilities for career progression that were being created. The socially constructed evolution of the GPN role had been one of the unintended consequences of the drive to move the emphasis from secondary to primary care. The new emergent CoP meant that students were able to appreciate the role in its social context, and see the various opportunities that presented themselves.

*“I think the placement* [in general practice] *helped me see that there are all different levels of nurses in primary care, not just the ones that do* [the] *simple stuff in returns clinics* [e.g. dressings]*, but the ones that do the more difficult stuff and have more responsibility* [such as] *the nurse practitioners who are like independent prescribers and do their own clinics and that...”*

(Interview S10)

Interestingly, some of the students contrasted their general practice experience with that of working on a hospital ward. The frame of reference for ‘good’ and ‘bad’ placements was beginning to shift as the general practice CoP was being developed and the established CoP for secondary care placements was being negatively influenced.

*“… the idea of a job there is attractive at all sorts of levels* [for me]*… I really do like the independence they get* [in general practice] *they’re basically allowed to do almost anything they want… Whereas, in hospital, they’re restricted by what the consultant wants or what the ward sister wants …”*

(Interview S18)

Another student had the same idea but from a slightly different perspective. She made a rather pragmatic but totally understandable point about the current state and culture of acute hospital wards:

*“Having seen what X* [a GPN] *does and the quality time she can spend with her patients* […] *that’s what I want to do when I qualify* […] *I don’t want to end up slogging my guts out* (sic) *on a busy medical ward, that’s for sure…”*

(Interview S17)

Although rather inelegantly put perhaps, this last quote does place the situated learning experience in primary care into stark contrast with the harsh reality of the learning experience in secondary care. Through the CoP, hearts and minds may be beginning to change.

### Limitations of the study

There are a number of potential limitations to this study. Although saturation was reached during the data collection, the sample of students interviewed was by its very nature limited to the population of those students who had experienced a general practice placement. In addition, the students who did agree to be interviewed were self-selecting and may no therefore represent an accurate cross-section of the student opinion. Although it was made clear to the students during the process of gaining informed consent that they would not be disadvantaged by taking part, the inevitable power dynamic between staff and student may also affect the veracity of the data.

## Discussion

In order to better appreciate the context in which this cultural shift is currently taking place, and to better guide the development of general practice as a suitable learning environment for student nurses, it may be useful to consider the ATPS in terms of an embryonic CoP [18, 19]. From a pedagogical perspective, it may be argued that students learn about general practice through participating in the shared social practices of the often arcane world of general practice, and their learning will be inextricably linked to the situated, contextual and social engagement that occurs within the general practice team. It may be argued that the students’ learning is therefore seen as a process in which they learn and develop into a proto-GPN, through the adoption of a new, albeit temporary identity. This provides the opportunity for them to positively influence the social context in which they are learning [18, 19]. This social and contextual learning is as equally valid for the GPs and the GPNs that the students come into contact with, and will be dealt with in more detail in a separate paper. Interestingly the results from this study are consistent with a number of studies from medicine [15, 16] which have looked at the career intentions of medical students. These studies identified the importance of positive exposure to the general practice environment, the need for good role models and a nurturing environment in which to participate in holistic, patient-centred care. This study significantly builds upon these findings by providing a working theoretical construct in which to contextualise and explain the findings.

Traditionally there has been little or no incentive for newly qualified graduate nurses to even consider applying for a GPN post [5]. This situation has arisen, at least in part, because GPs are small businesses. GPNs are employed by the GPs and not the NHS, and are therefore an added ‘cost’ to the GP business. Given that GPNs contribute a significant amount of income to the practice through meeting Quality Outcomes Framework (QOF) targets [8, 9], GPs prefer to recruit already experienced nurses, largely from secondary care, rather than invest in newly qualified graduate nurses and the extra costs involved in providing them with the education and training required for the GPN role [14]. When a GPN post has become vacant, there is also evidence of a GPN recruitment ‘merry go round’ in which new GPNs are often appointed by being poached from other GP practices locally [13, 14]. The continued recruitment of experienced nurses from either GP practices or other clinical environments has also meant that there has been no established career structure or pathway for neophyte GPNs to follow.

This situation has given rise to a number of ‘urban myths’. For example, graduating student nurses are often under the (mis)impression that they ‘need’ to have secondary care experience before applying for a GPN post [1, 13, 14]. These myths which were prevalent within the students’ narratives have had the effect of both dissuading graduate nurses from considering a career in general practice, and continuing to excuse GPs from employing them. As with most myths, they have assumed a certain degree of truth, and a negative spiral has resulted in which there is no place in general practice for new graduate nurses. The consequence of this negative spiral is that general practice has, in the past, been perceived by students and lecturers alike as a clinical ‘backwater’ that is not suitable for innovative, proactive new graduate nurses. The RCGP ‘Roadmap to Excellence’ report [3] clearly articulated the need to attract more new graduate nurses into general practice if the predicted increases in workload and complexity of care are to be satisfactorily addressed.

As part of the negative spiral, there was a perceived lack of time devoted to general practice nursing in the undergraduate nursing curriculum. This was further reinforced, albeit inadvertently, by the dearth of general practice clinical placements. The reality is that much of the students’ placement exposure in the past has been in the hospital setting, and there is still a widespread (if ill-informed) view that the majority of the students’ placement time should still be spent working on hospital wards. This has meant that student nurses have only ever spent an ‘odd’ day visiting a general practice, usually as a ‘taster’ day as part of a wider community nurse placement. This fleeting exposure to general practice has done little to positively influence the views of the students, as the time was often spent ‘sitting in’ with a GP during a routine clinic. As a consequence, the students have had up until now little, if any practical experience of the GPN role and what it currently involves or the opportunity to develop a CoP in general practice. In spite of some initial reservations, the CoP that has emerged from the ATPS has enabled the benefits of a general practice placement to filter through, albeit rather slowly and to limited numbers of students.

With the emergence of any new work-based culture, it is important to know how best to both cultivate and support a CoP. Simply bringing together a group of disparate staff and calling them a CoP does not mean that they will function as one [20]. A CoP is as vulnerable to dysfunctional relationships e.g. cliques as any other group, therefore it is vital that these are avoided. There are factors that will both influence and hinder the successes of these groups. One factor that needs to be taken into account is the use of social media. The ‘millennial’ Generation Y and Z students’ use of social media is widespread [22] and it is understandable and inevitable that social media will be used to obtain knowledge and information regarding these new placements. From a theoretical perspective, the use of social media by students may be seen as the first tentative steps in the development of a new CoP. As we are aware, students’ views of a placement are guided by information gleaned from social media sites such as Facebook^©^, however there remains a degree of ambivalence amongst academic staff over its use [22]. This ambivalence may be seen as a missed opportunity to positively influence the development of a new CoP or modify an existing one. Anecdotally, almost all our students are characterised as ‘active’ users of both Facebook^©^ and WhatsApp^©^, and whilst this is not actively encouraged, it is certainly not formally discouraged. Given that smart phones are an integral part of most people’s life, there is a strategic need to embrace their use, and to consider how best to embed social media into student learning and the development and maintenance of a CoP.

It is clear from the findings that the students’ perceptions of the current GPN role and how it ‘fits in’ to the wider healthcare context were based upon inaccurate, outdated information. Given that general practice has historically been peripheral to the placement experiences of most nursing students, it is perhaps not surprising that there is an identifiable need for more open dialogue and a greater understanding of ‘what makes each other tick’. Under the auspices of the ATPS, the development of the general practice CoP will go some way to address these issues. From a knowledge perspective, GPs are clearly familiar and comfortable with the development needs of medical students and the GP trainees they support, but much less familiar or comfortable with the learning needs of both the GPNs they employ and the student nurses that are being placed with them. In addition, since GPNs are recruited and employed by the GP partners who own the business, there has been little incentive to change the culture of GPN recruitment and education [23].

An appreciation, through a CoP, of the socially constructed nature of general practice will provide an opportunity for the cultures of both general practice and nurse education to evolve together and to better reflect the new ways of working advocated by the ‘Five Year Forward View’ and being delivered through the NTHI and the ‘10 point plan’. Once the CoPs become well-established within general practice, this will enable the neophyte GPNs that enter general practice to develop their own work-based identity, which will in turn provide the impetus for future GPN role development.

## Conclusions

By facilitating the development of a Community of Practice (CoP) for student nurses within general practice comparable to those provided within the hospital-based setting, the students were able to gain a much more authentic insight into general practice and the role of the GPN. The GPN role, as it has developed, is an example of autonomous, patient-focused care. This is particularly apparent for the care of individuals with long term conditions and was evident from the vast majority of the students’ interviews. There is a clear need to pursue and embed the cultural changes initiated by schemes such as the ATPS, so that newly qualified graduate nurses may be both recruited into general practice and provided with a career structure equivalent to that found elsewhere. Having experienced and contributed to the ‘community of general practice’, there is clearly a cohort of students who would like a career in general practice, and it is to be hoped that the opportunities presented by the ATPS will continue to be supported and nurtured. If the much-discussed workforce crisis in general practice is to be averted, then schemes such as this are crucial to increasing the numbers of GPNs. This study indicates that the ATPS placements are helping to promote a change in the culture and practice of GPN recruitment and retention, and a new philosophy of ‘growing your own’ GPNs is now gaining some traction. The CoP has slowly and subtly begun to change the entrenched attitudes of both general practice and student nurses towards the idea of new graduate nurses working and having a career in a general practice setting.

## Additional file

Additional file 1: Interview schedule student.doc. A copy of the interview schedule used for the interviews with the students. (DOCX 14 kb)

## Abbreviations

ATPS: Advanced Training Practice Scheme
CCG: Clinical Commissioning Group
CoP: Community of Practice
CPEN: Community Education Provider Network
GP: General Practitioner
GPN: General Practice Nurse
HEE: Health Education England
HEI: Higher Education Institution
NHS: National Health Service
NTHI: National Training Hubs Initiative
QNI: Queen’s Nursing Institute
RCGP: Royal College of General Practitioners (UK)
SHU: Sheffield Hallam University
UK: United Kingdom
