# Supplementary material for: Changing hearts and minds: examining student nurses’ experiences and perceptions of a general practice placement through a ‘community of practice’ lens
Source: BMC Med Educ. 2018 Apr 5;18:67. doi: 10.1186/s12909-018-1182-6 (PMC5887172; doi:10.1186/s12909-018-1182-6)
Supplement: Supplementary file 1 — Interview schedule student.doc. A copy of the interview schedule used for the interviews with the students. (DOCX 14 kb) [file 12909_2018_1182_MOESM1_ESM.docx]

**Interview schedule: student nurses**

1. Introductions and preamble
2. Have you had a clinical placement in general practice? Which year were you in?
3. What did you know about general practice before you started? Where did that information come from?
4. What were your thoughts before going out on the placement?
5. How does general practice 'work'? What do GPNs do?
6. What are your thoughts about the placement now?
7. Have your views changed following the placement? Please can you expand upon your answers?
8. Would you consider a career in general practice? Please can you expand upon your answers?
